# Persistent multifocal pulmonary consolidations in a young adult: An unusual presentation of primary pulmonary follicular lymphoma

**DOI:** 10.1016/j.rmcr.2026.102516

**Published:** 2026-09-29

**Authors:** Thitapa Luepiyapanich, Komsai Suwanno, Putachart Misang

**Affiliations:** a Division of Respiratory Medicine, Department of Medicine, Hatyai Hospital, Songkhla, Thailand; b Division of Hematology, Department of Medicine, Hatyai Hospital, Songkhla, Thailand; c Department of Pathology, Hatyai Hospital, Songkhla, Thailand

## Abstract

Primary pulmonary lymphoma (PPL) is rare malignancies, accounting for less than 1% of primary lung malignancies and extranodal lymphomas. Primary pulmonary follicular lymphoma is an uncommon subtype of PPL. Herein, we report the case of a 29-year-old immunocompitent man who presented with persistent pulmonary infiltrations despite appropriate antibiotic treatment for presumed pneumonia. Computed tomography (CT) of chest revealed multifocal consolidations with surrounding ground-glass opacities, predominantly involving the peripheral and lower lung zones, along with multiple mediastinal lymphadenopathy. Brochoscopy with bronchoalveolar lavage cultures and microbiological investigation, including tests for bacterial, mycobacterial, and fungal pathogens, were all negative. Histological examination of transbronchial biopsy (TBBx) demonstrated atypical B-cell lymphoproliferation, Immunohistochemistry was positive for CD20, CD10, BCL2, and BCL6, while AE1/AE3 and thyroid transcription factor-1 (TTF1) highlighted reactive pneumocytes. No evidence of any extrathoracic manifestration is comfirmimg the diagnosis of primary pulmonary follicular lymphoma. The patient was treated with rituximab, cyclophosphamide, vincristine, and prednisolone (R-CVP), resulting in significant clinical and radiographic improvement. This case highlights the diagnostic challenge of primary pulmonary follicular lymphoma, which may mimic chronic pulmonary infection, particularly in young immunocompetent patients. Persistent pulmonary consolidations despite appropriate antimicrobial therapy should prompt consideration of early tissue diagnosis to exclude an underlying malignancy.

## 1 Introduction

Primary pulmonary lymphoma (PPL) is rare extranodal lymphoma confined to lung parenchyma or bronchi, with or without mediastinal lymph node involvement, and without evidence of extrathoracic disease at the time of diagnosis or within three months thereafter. It accounts for less than 1% of all primary pulmonary malignancies and approximately 3.6% of extranodal lymphomas [[1], [2], [3]]. PPL typically occurs in individuals in the fifth to seventh decades of life and is more frequently reported in immunocompromised patients, particularly recipients of solid organ transplantation. Nevertheless, it can also occur in immunocompetent patients.

The majority (70-80%) of PPL are mucosa-associated lymphoid tissue (MALT) lymphomas, whereas primary pulmonary follicular lymphoma is an extremely rare histological subtype. To date, most reported cases of primary pulmonary follicular lymphoma have been described as case reports or small case series [4]. Furthermore, its clinical and radiological manifestations are often nonspecific and may closely resemble chronic infections or inflammatory lung diseases, resulting in delayed diagnosis.

Herein, we report a case of primary pulmonary follicular lymphoma in 29-year-old immunocompetent man who present with persistent pulmonary infiltrations despite appropriate antibiotic therapy for presumed pneumonia. This case highlights the importance of considering pulmonary lymphoma in the differential diagnosis of non-resolving pneumonia, even in young patients without apparent immunosuppression.

### 1.1 Case presentation

#### 1.1.1 Clinical presentation

A 29-year-old man presented to the outpatient clinic with a two-months history if intermittent pleuritic chest pain. He denied cough, dyspnea, hemoptysis, fever, weight loss, fatigue, or night sweats. He initially sought medical attention at a primary care hospital, where he was treated empirically with oral amoxicillin-clavulanate for 10 days for presumed community-acquired pneumonia. Despite antibiotic therapy, his symptoms persisted, and follow-up chest radiography demonstrated progressive pulmonary infiltrates. He was therefore referred to our tertiary care center hospital for further evaluation.

His past medical history was unremarkable. He had no history of smoking, illicit drug use, or regular medication use. He was born and raised in the southern of Thailand and reported no history of closed contacted with patients with tuberculosis, recent travel, occupational exposure, or a family history of malignancy.

#### 1.1.2 Physical examination

On presentation, the patient appeared well nourished and was in no acute distress. His vital signs were within normal limits, with a body temperature of 36.8^°^C, respiratory rate of 18 breaths/min, Heart rate 80 beats/min and oxygen saturation of 97% while breathing on room air. Respiratory examination demonstrated normal chest expansion with normal breath sound bilaterally and no adventitious sounds. The remainder of the physical examination was unremarkable.

#### 1.1.3 Diagnostic studies

Initial laboratory tests were as follows: hemoglobin 14 g/dl, total white blood cell count of 7300/μL (neutrophils 58%, leukocytes 34%), platelet count of 216,000/μL BUN 12 mg/dl, Cr 1.05 mg/dl, albumin 4.7 g/dl, globulin 2.2 g/dl, AST 24 U/L, ALT 9 U/L and ALP 57 U/L. Immunodeficiency was ruled out by negative anti-HIV.

Chest radiography demonstrated progressive bilateral alveolar infiltrates, predominantly involving the lower lung zones compared with imaging obtained one month earlier (Fig. 1A–B). Contrast-enhanced chest computed tomography (CT) demonstrated multifocal consolidations with surrounding with ground-glass opacities, in a predominantly lower-lobe distribution. Several lesions exhibited a flame-shaped like pattern. Multiple enlarged mediastinal lymph nodes, including parathracheal and prevascular nodes, were also identified (Fig. 2).

**Fig. 1:**
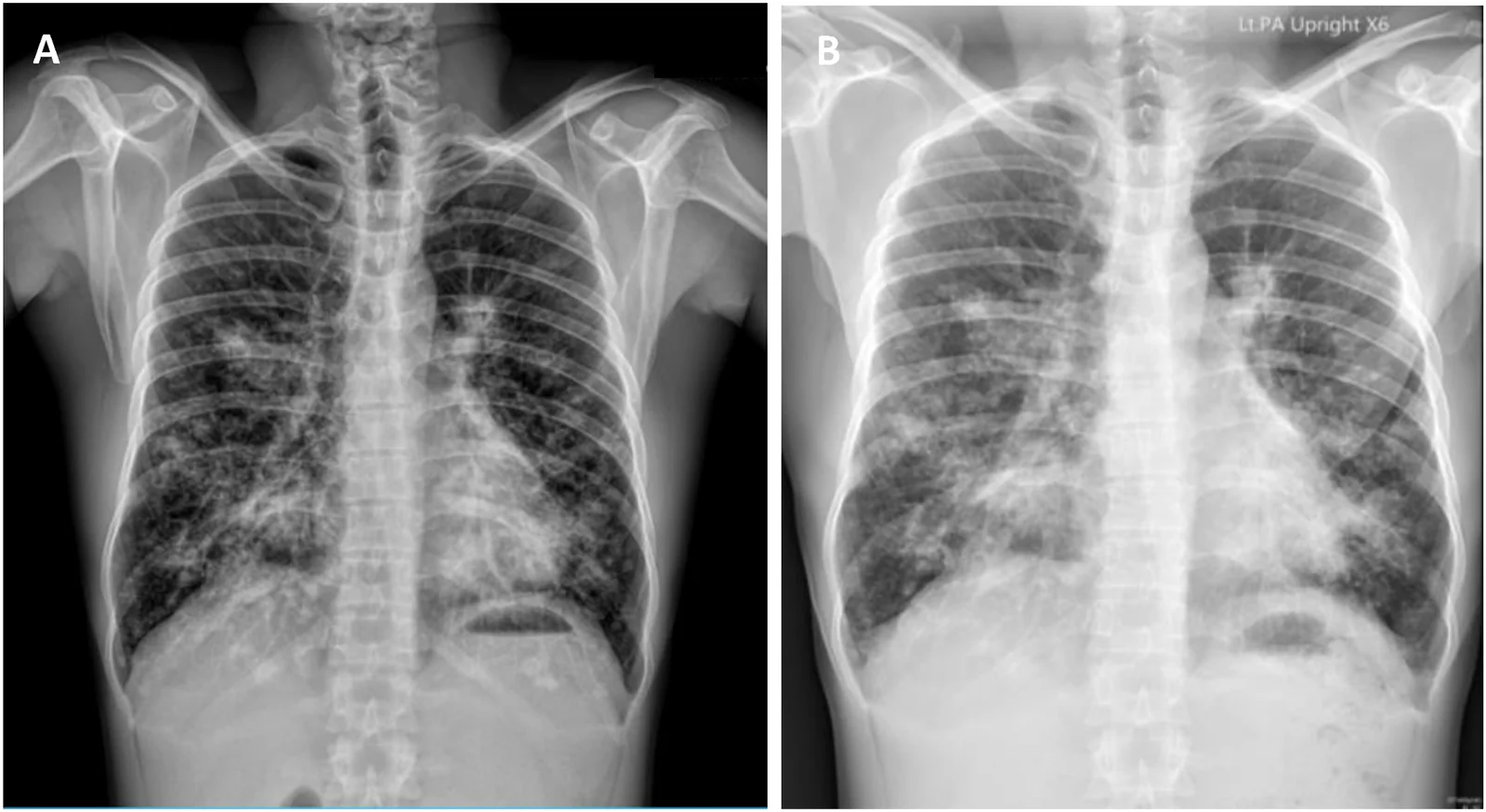
A: CXR at primary care hospital showed bilateral alveolar, predominant lower lung zone. B: CXR first visit at OPD demonstrated increase alveolar infiltration at both lungs.

**Fig. 2:**
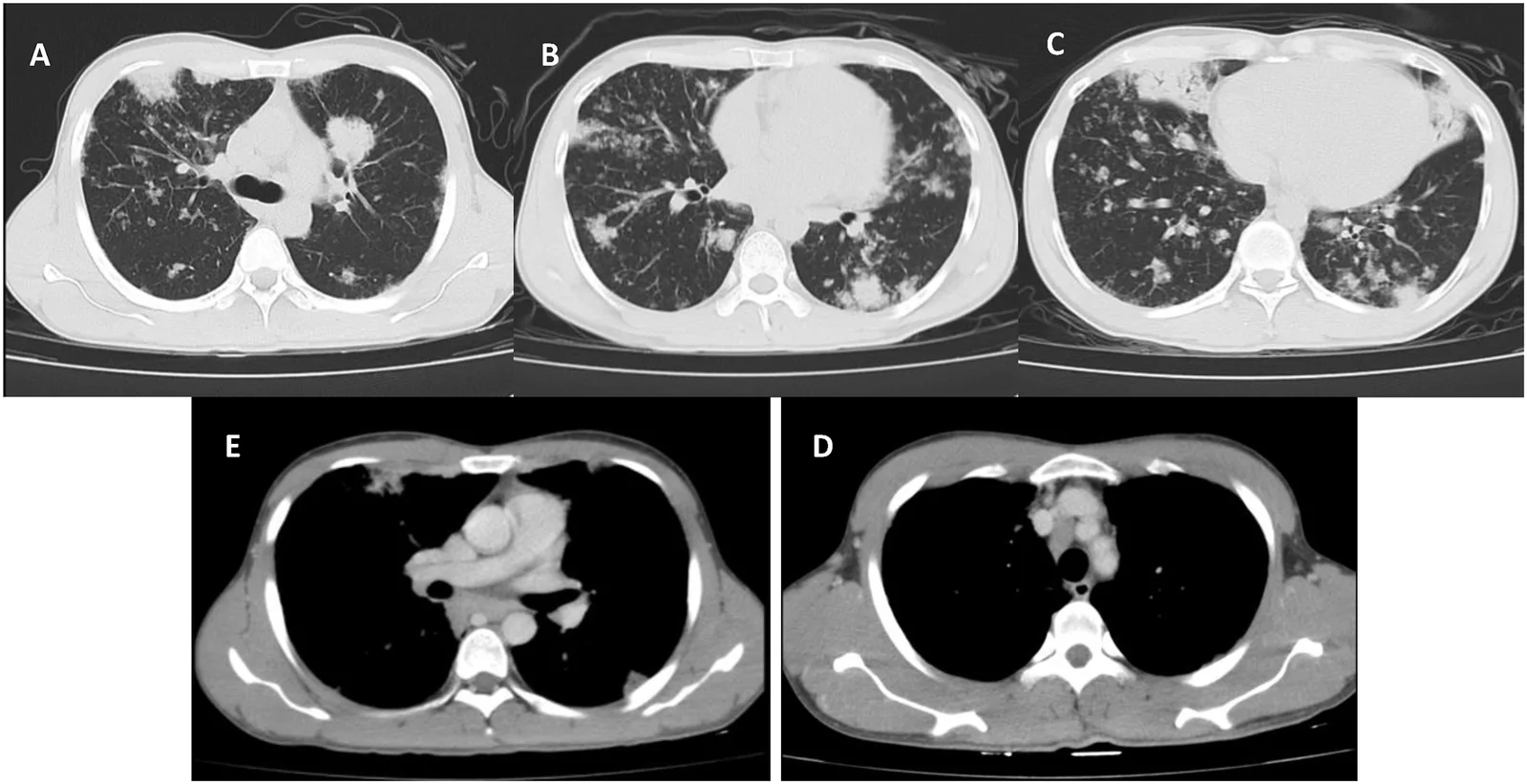
A-C: CT chest in lung window showed multiple consolidation with ground-glass opacities scattering both lung. D-E: CT chest in mediastinal window showed multiple lymphadenopathies (star marker).

Flexible bronchoscopy with bronchoalveolar lavage (BAL) and transbronhcial lung biopsy (TBBx) was performed. Gram stain, acid-fast bacilli (AFB), modified AFB and galactomannan assay of BAL fluid were all negative. BAL cultures for bacteria, mycobacteria, fungal pathogens were also negative. Cytologic examination demonstrated no malignant cells.

Histopathologic examination of transbronchial biopsy specimens demonstrated atypical lymphoid infiltrates composed of B-cells. Immunohistochemical staining showed positively for for CD20, CD10, BCL2, BCL6, and CD10, while AE1/AE3 and thyroid transcription factor-1 (TTF1) highlighted reactive pneumocytes (Fig. 3). These findings were consistent with follicular lymphoma. Special stain for acid-fast bacilli (AFB) and fungi (GMS) were negative.

**Fig. 3:**
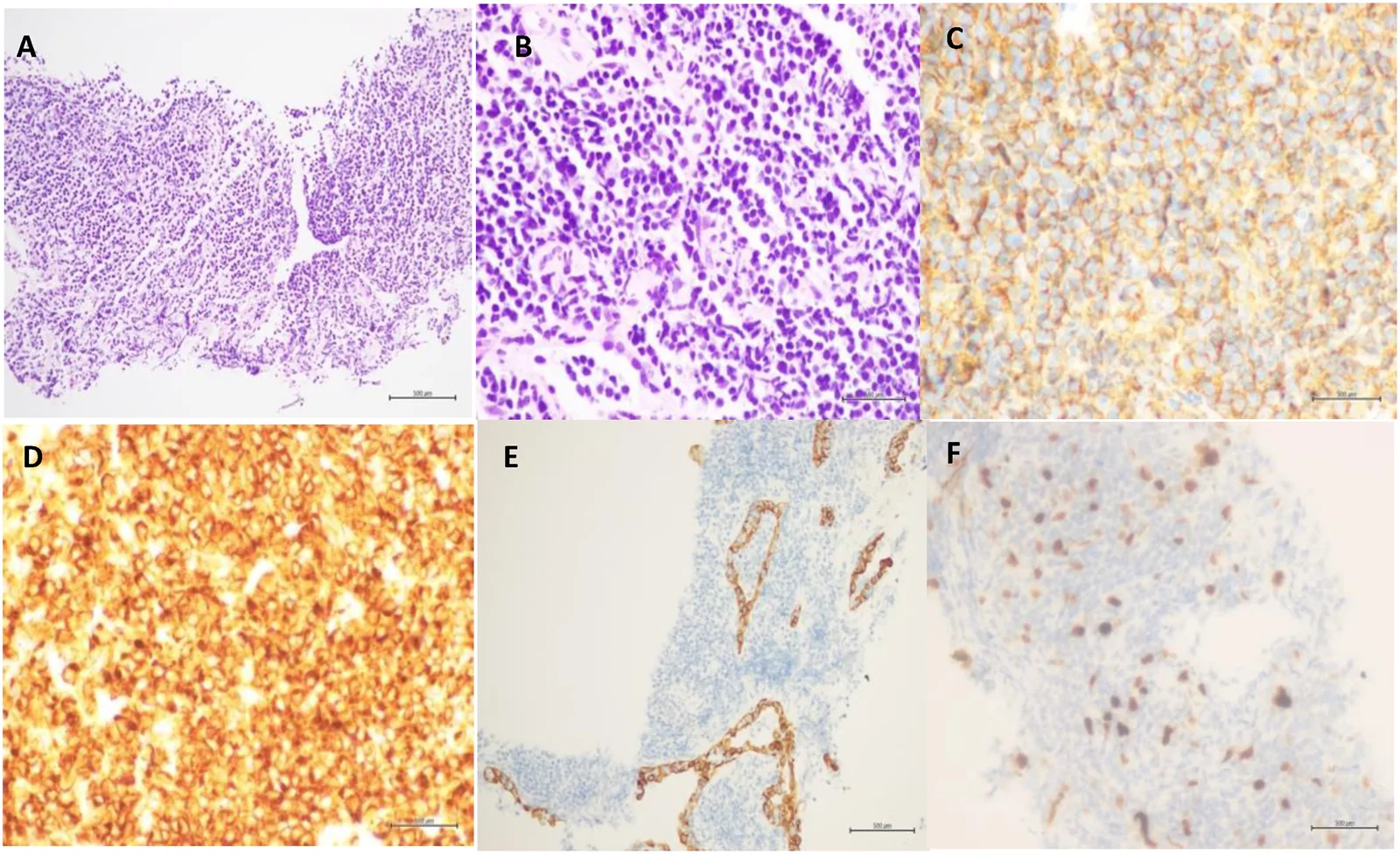
A-B: H&E stain (x20 and ×60 magnification) lung was architectural effacement by diffuse proliferation of atypical lymphocytes. C: strongly positive for CD20 (x60 magnification) D: strongly positive for BCL2 (x60 magnification) E: positive AE1/AE3 (x20 magnification) F: The Ki67 labelling index was 20% (x40 magnification) Overall, the histomorphology and Immunohistochemical results suggested that the lesion was follicular lymphoma, grade 1.

Contrast-enhancing CT of abdomen demonstrated no evidence of extrathoracic disease. Bone marrow biopsy revealed normocellular marrow without lymphomatous involvement. Collectively, these findings fulfilled the diagnostic criteria for primary pulmonary follicular lymphoma.

#### 1.1.4 Treatment and follow up

The patient received combination chemotherapy consisting of rituximab, cyclophosphamide, vincristine and prednisolone (R-CVP). After three cycles of chemotherapy, his pleuritic chest pain completely resolved. Follow-up CT of chest demonstrated markedly decreased multiple consolidations and decreased size of mediastinal lymph nodes; no additional abnormalities were identified. The treatment plan consisted of a total of six cycles of chemotherapy, followed by up clinical follow-up and repeat imaging for response assessment.

## 2 Discussion

Primary pulmonary lymphoma (PPL) is a rare extranodal lymphoma that predominantly affects in middle-age to elderly individuals. Patients commonly present with nonspecific symptoms such as cough (approximately 50%), dyspnea (40%) and pleuritic chest pain (17%), although up to one-third of patients may be asymptomatic and diagnosed incidentally on chest imaging [2,3]. Radiological findings are highly variable, ranging from solitary or multiple pulmonary nodules and mass-like consolidations to diffuse infiltrates, cavitary lesions or pleural effusions, with or without mediastinal lymphadenopathy. Routine laboratory investigation are often nonspecific, and serum lactate dehydrogenase (LDH) levels may remain within the normal range, particularly in indolent histological subtypes such as follicular lymphoma [4,5]. Consequently, establishing an early diagnosis based solely on clinical, radiological, and laboratory finding is often challenging.

Given these nonspecific features, the differential diagnosis of PPL is broad and includes chronic infections, particularly tuberculosis and fungal infections, primary lung carcinoma, metastatic malignancy and inflammatory or autoimmune conditions such as sarcoidosis, vasculitis and rheumatoid nodules. In young immunocompetent patients, infectious and inflammatory etiologies are generally considered more likely than lymphoma, which may contribute to delayed diagnosis. In this present case, the patient's young age, absence constitutional symptoms or immunosuppression, and imaging findings of multifocal pulmonary consolidations initially favored an infectious etiology. However, persistent radiographic abnormalities despite appropriate antimicrobial therapy and negative microbiological investigations prompted further histological evaluation, ultimately establishing the diagnosis of primary pulmonary follicular lymphoma.

To better characterized the clinical spectrum of primary pulmonary follicular lymphoma, we reviewed previously published cases in the English-language literature (Table 1). Most reported patients were older than 50 years old, and their clinical presentation and radiological finding were heterogeneous, ranging from asymptomatic solitary pulmonary nodule to diffuse pulmonary infiltrates and pleural effusions. In contrast, our patient was lonely 29 years old and presented with persistent multifocal pulmonary consolidations despite appropriate antibiotic treatment, highlight an uncommon presentation of this rare disease.

**Table 1:** Clinical spectrum of primary pulmonary follicular lymphoma.

Author	Year	Age	Sex	Symptoms	CT	Treatment
Takuma K et al. [6]	2019	62	female	asymptomatic	Bilateral diffuse micronodules	-
Terada T [7]	2011	50	female	Productive cough	Single nodule	Wedge resection
Luis GM et al. [8]	2022	78	male	dyspnea	Fibrosis with traction bronchiectasis (UIP pattern)	-
Sarah EK et al. [9]	2024	74	male	asymptomatic	Single nodule	Wedge resection
Panchal M. et al. [10]	2024	74	male	Dyspnea, fever and weight loss	Bilateral pleural effusion	-
Present case	2025	29	Male	Pleuritic pain	Bilateral multiple consolidation with GGO	R-CVP

This case emphasizes the importance of considering primary pulmonary lymphoma in the differential diagnosis of non-resolving pneumonia, even in young immunocompetent patients. Failure of pulmonary infiltrates to improve after appropriate antimicrobial therapy, particularly in the setting of repeatedly negative microbiological investigations, should prompt early tissue sampling to establish a definitive diagnosis. Early recognition may facilitate timely initiation of appropriate therapy while avoiding unnecessary investigations, prolonged empirical antimicrobial treatment, and delays in definitive diagnosis.

## 3 Conclusion

Primary pulmonary follicular lymphoma is an uncommon cause of persistent pulmonary consolidations and may closely mimic chronic pulmonary infection, even in young immunocompetent patients. Failure to respond to appropriate antimicrobial therapy, together with negative microbiological investigations, should prompt early histopathological evaluation. Awareness of this rare entity may facilitate timely diagnosis, appropriate treatment, and favorable clinical outcomes while avoiding unnecessary delays associated with prolonged empirical therapy.

## CRediT authorship contribution statement

**Thitapa Luepiyapanich:** Conceptualization, Data curation, Project administration, Writing – original draft. **Komsai Suwanno:** Conceptualization, Investigation, Writing – review & editing. **Putachart Misang:** Writing – review & editing.

## Declaration of competing interest

The authors declare that they have no known competing financial interests or personal relationships that could have appeared to influence the work reported in this paper.
